# A Rare Case of Acute Lymphoblastic Leukemia in a Four-Month-Old Infant

**DOI:** 10.7759/cureus.86741

**Published:** 2025-06-25

**Authors:** Noora AlSuwaidi, Razan Ismail, Bosaina Otour

**Affiliations:** 1 Pediatric Emergency Medicine, Al Jalila Children's Specialty Hospital, Dubai, ARE

**Keywords:** b-cell acute lymphoblastic leukemia, hepatomegaly, infant, leukemia, lymphoblastic leukemia

## Abstract

Acute lymphoblastic leukemia (ALL) is the most common pediatric malignancy, yet its presentation in infants under one year is rare and often atypical. This report describes a four-month-old female infant who presented with fever, vomiting, and pallor, ultimately diagnosed with precursor B-cell ALL. Laboratory investigations revealed severe anemia, thrombocytopenia, and hyperleukocytosis, with 95% circulating blasts. Immunophenotyping confirmed the diagnosis, and further cytogenetic studies were recommended. This report emphasizes the need for early recognition of clinical and laboratory signs, such as hyperleukocytosis, severe anemia, and a high burden of circulating blasts in infants, to facilitate timely diagnosis and initiation of risk-adapted treatment strategies for ALL.

## Introduction

Leukemia constitutes approximately one-third of pediatric cancers, with acute lymphoblastic leukemia (ALL) being the most prevalent subtype. While pediatric leukemia is relatively common, its occurrence in infants, particularly those younger than six months, remains rare and diagnostically challenging [1]. Infants (<1 year) represent approximately 2-5% of ALL cases and are considered a distinct biological subgroup characterized by unique genetic abnormalities, such as mixed-lineage leukemia (MLL) rearrangements, and significantly inferior prognosis compared to older children [1,2]. Unlike older children, infants with ALL often present with non-specific symptoms, high white blood cell (WBC) counts, and aggressive disease features including hepatosplenomegaly, leukemia cutis, and early central nervous system (CNS) involvement [3]. This report presents a four-month-old female infant diagnosed with ALL, focusing on the diagnostic process, management considerations, and prognostic implications unique to this age group.

## Case presentation

A previously healthy four-month-old female infant presented to the pediatric emergency department with a four-day history of fever, vomiting, and reduced activity. The mother reported a similar admission at one month of age for fever and poor feeding, during which preliminary investigations - including hemoglobin and white blood cell count - were reportedly normal, and the infant was discharged after symptomatic management. However, documentation from that visit was unavailable for full comparison. This prior febrile episode may have represented an early hematologic aberration or an infectious trigger, potentially contributing to leukemic progression.

There was also recent exposure to a mildly ill sibling with an upper respiratory tract infection, though the temporal proximity and the patient’s immunocompromised state raise the possibility of infection as a contributory stressor. On examination, the infant appeared pale, with hepatomegaly and a soft systolic murmur. Her vital signs were as follows: temperature 37.1°C, heart rate 163 bpm, respiratory rate 38 breaths/min, blood pressure 88/62 mmHg, and oxygen saturation of 96% on room air.

Initial laboratory investigations revealed severe anemia, marked leukocytosis, and thrombocytopenia (Table 1). A peripheral smear showed 95% circulating blasts with high nuclear-to-cytoplasmic ratios, prominent nucleoli, and fine chromatin. Flow cytometry confirmed precursor B-cell acute lymphoblastic leukemia (B-ALL), with the blasts expressing CD123, CD73/86, CD9, CD34, TdT, HLA-DR, CD58, CD38, cCD22, sCD22, cCD79a, and CD19, along with partial expression of CD24 and aberrant CD33.

**Table 1:** Summary of initial laboratory investigations demonstrating pancytopenia and hyperleukocytosis.

Investigations	Results	Normal range
Hemoglobin	4.0 g/dL	10.5-13.5 g/dL
White blood cells	899.5×10³/µL	5-19×10³/µL
Red blood cells	1.53×10⁶/µL	3.1-4.5×10⁶/µL
Platelets	25,000/µL	150,000-450,000/µL
Lactate dehydrogenase	1,803 U/L	Varies by laboratory
Uric acid	8.8 mg/dL	2.5-5.5 mg/dL
Phosphorus	4.3 mg/dL	4.5-6.5 mg/dL
Peripheral smear	95% blasts, high nuclear-to-cytoplasmic ratio, prominent nucleoli	No blasts
Flow cytometry	Precursor B-cell acute lymphoblastic leukemia markers; CD19+, CD22+, terminal deoxynucleotidyl transferase (TdT)+, CD34+, CD123+, partial CD24+, aberrant CD33+	No abnormal expression
Cytogenetics	Sent for mixed-lineage leukemia (MLL) gene rearrangement and risk stratification	No MLL rearrangement

Cytogenetic and molecular studies were performed to evaluate for high-risk features, particularly rearrangements involving the KMT2A gene (formerly MLL), which are frequently seen in infant ALL and are associated with poor prognosis. At the time of writing, KMT2A rearrangement status and next-generation sequencing (NGS) results were pending, with plans to use these findings to guide risk stratification and determine the need for hematopoietic stem cell transplantation (HSCT). RNA fusion panels and molecular profiling are also underway to detect potential leukemia-related mutations.

The patient was initiated promptly on an infant-adapted chemotherapy protocol consistent with modified Children’s Oncology Group (COG) guidelines. Blinatumomab was not used in the induction phase. A central venous catheter was inserted to facilitate chemotherapy delivery and laboratory monitoring. Supportive care included packed red blood cell transfusions (10 mL/kg infused slowly over 4 hours), platelet transfusions to maintain counts >50,000/µL, and IV hydration. Rasburicase was administered for tumor lysis management due to pre-existing hyperuricemia and elevated LDH, while allopurinol was avoided given the severity of laboratory tumor lysis. CNS evaluation via lumbar puncture was deferred until leukocyte counts decreased due to the risk of herniation. Initial bone marrow aspirate revealed hypercellularity with >90% blasts.

The infant tolerated the initial phase of chemotherapy without early complications. Her vital signs stabilized, and transfusion requirements decreased. Post-induction response, including measurable residual disease (MRD) status and day 29 marrow evaluation, is pending at the time of report submission. The hematology/oncology team continues to closely monitor for treatment response and chemotherapy-related toxicities.

## Discussion

Clinical presentation and diagnostic considerations

Infant ALL often presents with vague symptoms that overlap with common pediatric infections or hematologic disorders [1,3]. In this case, initial concerns were limited to fever and vomiting. However, the infant’s pallor, hepatomegaly, and laboratory profile (severe anemia, thrombocytopenia, hyperleukocytosis) prompted further investigation. Emergency physicians should maintain a high index of suspicion for leukemia when infants present with unexplained pallor, organomegaly, failure to thrive, or persistent fever, particularly in the setting of laboratory derangements.

In infants, distinguishing ALL from other causes, such as transient myeloproliferative disorder (particularly in neonates with Down syndrome), transient abnormal myelopoiesis (TAM), or congenital leukemia, requires urgent immunophenotyping and cytogenetic testing to avoid diagnostic delay. Peripheral blood smear and flow cytometry remain essential for identifying leukemic blasts and establishing immunophenotype. The patient’s immunoprofile was consistent with precursor B-cell ALL [1,2]. The extremely high WBC count (hyperleukocytosis) raises concern for leukostasis and early complications, such as CNS involvement and respiratory distress, events more common in infants, and necessitating rapid intervention and supportive care.

A study by Zhang et al. analyzing 59 congenital leukemia cases found leukemia cutis in 67.8% of patients, CNS involvement in 25.4%, and hepatosplenomegaly in 47.5% of patients [4]. Hyperleukocytosis, defined as WBC >100,000/µL, was present in over 60% of cases and is associated with increased early mortality risk [4].

Given the patient’s age and severity of presentation, clinicians appropriately pursued molecular testing and cytogenetics. These tests guide risk stratification and may identify high-risk genetic alterations, such as KMT2A (formerly MLL) rearrangements, found in over 70% of infant ALL cases and associated with a poor prognosis [3].

Etiology and pathophysiology

The pathogenesis of infant leukemia involves a prenatal genetic lesion - frequently MLL (KMT2A) gene rearrangement - combined with postnatal events, such as epigenetic dysregulation, aberrant immune responses to infection, or additional somatic mutations that trigger full leukemogenesis [5,6]. This “two-hit” model accounts for the aggressive biology and early onset of infant ALL.

In this case, the earlier febrile episode at one month of age may reflect early hematologic disruption or an immunological trigger contributing to disease acceleration. The rapid clinical progression and the immunophenotype support a prenatal origin with subsequent transformation.

Treatment considerations

Treating infant ALL requires significant deviation from standard pediatric regimens due to the infant’s altered physiology and high-risk disease biology [1,6]. Infants under six months have unique pharmacokinetics, increased sensitivity to chemotherapy-related organ toxicities, and heightened susceptibility to infections, necessitating customized approaches.

Protocols, such as Interfant-21 and modified Children’s Oncology Group (COG) regimens, are often employed. These regimens are intensified and include adjusted dosing based on pharmacokinetic parameters, CNS prophylaxis through intrathecal therapy, and early response-guided risk stratification, especially crucial given the higher relapse rates in infants with KMT2A rearrangements.

Supportive care, such as transfusions, infection prophylaxis, and tumor lysis syndrome management, is critical during initial treatment phases. In this patient, transfusions were administered judiciously due to the risk of hyperviscosity, and rasburicase was used, given laboratory evidence of tumor lysis syndrome. Chemotherapy was delivered via central venous access, with close monitoring for organ toxicity.

Prognosis

Infant ALL is associated with significantly poorer outcomes compared to older pediatric populations, primarily due to aggressive disease biology, treatment-related toxicity, and early disease burden. Historically, the event-free survival (EFS) rate in high-risk infants, particularly those with KMT2A (MLL) rearrangements, has ranged from 30 to 50% [3,6].

However, recent data from large cooperative studies show a modest but meaningful improvement. The Interfant-06 study demonstrated improved EFS exceeding 40%, and the addition of blinatumomab to post-induction therapy has shown promising results, with two-year disease-free survival rates reaching up to 81.6% in select patient populations. These advances highlight the potential of immunotherapy and response-adapted strategies in improving outcomes for infant leukemia.

Long-term prognosis improves with early diagnosis, individualized chemotherapy protocols, and, when indicated, hematopoietic stem cell transplantation (HSCT). Nonetheless, survival remains guarded in infants presenting with hyperleukocytosis, CNS involvement, or unfavorable cytogenetics.

Similar to other hematologic malignancies that may present with subtle or non-specific symptoms, this case underscores the importance of maintaining a high level of clinical suspicion and ensuring timely escalation of care in infants with unexplained but concerning presentations.

## Conclusions

This case underscores the critical importance of considering leukemia in infants presenting with non-specific symptoms, such as pallor, fever, and vomiting, particularly when accompanied by alarming laboratory findings like hyperleukocytosis, severe anemia, and circulating blasts. In this four-month-old patient, prompt evaluation through flow cytometry enabled early diagnosis of precursor B-cell ALL. The use of an infant-specific chemotherapy protocol, adapted for the patient’s age and physiological vulnerability, facilitated safe induction and clinical stabilization.

Given the historically poor prognosis associated with infant ALL - especially in cases involving KMT2A rearrangements - timely diagnosis, molecular risk stratification, and tailored treatment are essential. While this report illustrates a typical clinical presentation and management pathway, emerging therapies such as blinatumomab, menin inhibitors, and response-adapted regimens offer promise for improving event-free and overall survival. Incorporating these advances into future treatment protocols may help overcome the historically guarded outcomes seen in this high-risk population.
